# Determinants of quality of life in women immediately following the completion of primary treatment of breast cancer: A cross-sectional study

**DOI:** 10.1371/journal.pone.0258447

**Published:** 2021-10-15

**Authors:** Jin-Hee Park, Yong Sik Jung, Ji Young Kim, Sun Hyoung Bae

**Affiliations:** 1 College of Nursing Research Institute of Nursing Science, Ajou University, Suwon, South Korea; 2 Department of Breast Surgery, School of Medicine, Ajou University, Suwon, South Korea; Chinese University of Hong Kong, HONG KONG

## Abstract

**Backgrounds:**

Many breast cancer patients experience significant distress immediately following the completion of primary treatment. Women who report low levels of quality of life (QOL) early in this phase of transitional survivorship tend to experience diminished long-term adjustment. However, since most of the prior studies on survivors were conducted on patients at various times, studies on QOL of women during the end of primary treatment have been insufficient. This study aimed to identify determinants of QOL in women with breast cancer immediately following the completion of treatment.

**Methods:**

A cross-sectional study was conducted on 140 disease-free breast cancer patients who had completed therapy in the past 1 month at university hospitals. Functional Assessment of Cancer Therapy-Breast (FACT-B), Memorial Symptom Assessment Scale-Short Form (MSAS-SF), Self-Efficacy Scale for Self-Management of Breast Cancer (SESSM-B), and Interpersonal Support Evaluation List-12 (ISEL-12) scales were used to assess predictors and QOL. The data were analyzed using the Pearson correlation, t-test, ANOVA, and hierarchical multiple regression.

**Results:**

The mean score of QOL for breast cancer survivors was 97.23 (±20.01). Chemotherapy and perceived economic status were significantly associated with QOL in terms of sociodemographic and disease/treatment-related characteristics. Physical and psychological symptoms and social support had a significant association with QOL. The regression analyses showed that physical and psychological symptoms and belonging support were statistically significant in predicting the QOL of breast cancer survivors.

**Conclusions:**

The variables of symptom experience and social support must be acknowledged when improving women’s QOL immediately after their completion of primary breast cancer treatment. Greater focus on the reduction of symptom distress and increasing a sense of belonging could improve QOL among breast cancer survivors.

## Introduction

Breast cancer is the second most common cancer among women, with a five-year survival rate of 93.2%. The incidence of breast cancer in Korea was 24.2% in 2018. The number of breast cancer survivors has been consistently increasing [1]. Whereas breast cancer commonly affects those aged 50 years or older in the United States and Europe, it has the highest incidence among those in their 40s in Korea. The earlier onset of breast cancer means a longer period lived as a breast cancer survivor. Attention has been drawn to issues regarding the quality of life (QOL) of breast cancer survivors [1]. In particular, the transition from patient to survivor may play a role in the QOL of breast cancer survivors; active attention is required to help patients resume their former lives and improve their QOL [2].

The successful completion of adjuvant treatment marks the beginning of the transitional survivorship phase, during which the patient transitions from ’’cancer patient’’ to ’’cancer survivor’’ [2, 3]. Patients with breast cancer who have completed their adjuvant treatment wish to return to their normal lives as soon as possible. Friends and family might also have unrealistic expectations about how quickly everyone concerned can return to their former lives. Survivors have the challenge of coping with multiple long-term side effects of treatment, both physically (e.g., pain, fatigue) and psychologically (e.g., anxiety, depression) [4], and are also at a higher risk of cardiovascular disease, lymphedema, and osteoporosis in the long term [5]. Therefore, patients with breast cancer who have completed treatment require intensive care for their physical and psychological symptoms [6]. However, dwindling attention and support from medical staff and family over time can lead to patients feel abandoned and fearful of possible recurrence and metastasis after they complete treatment [7]. Physical and psychological distress negatively affect overall QOL and hinder recovery in patients with breast cancer [7–9]. Therefore, it is necessary to understand the factors that affect QOL after completion of treatment to allow patients to successfully transition to cancer survivor status and learn to live with cancer [5, 7].

Major factors that affect breast cancer patients’ QOL after their completion of the primary treatment include physical and psychological symptoms [10–12], self-efficacy [13–15], and social support [16–18]. Symptom experience is an especially important factor in the QOL of patients with breast cancer. Many patients with breast cancer experience physical and psychological symptoms including depression, anxiety, poor body image, cognitive impairment, sleep disorder, pain, and fatigue [10–12]. As the distress from symptom experience increases, physical and social functioning and overall QOL decrease [10]. Physical and psychological symptoms experienced by patients with breast cancer are managed throughout the treatment period following a diagnosis. However, these symptoms persist for years even after the treatment is complete. Studies have emphasized the importance of symptom management for patients with breast cancer, since physical and psychological symptoms experienced after completion of treatment can hinder the patients’ return to normal life [11, 19].

Self-efficacy denotes the belief that one has the power to produce a desired effect by completing a given task or activity and the expectation that one can master a situation and produce a positive outcome [20]. Breast cancer survivors must continuously manage their health by ceasing smoking, reducing alcohol consumption, exercising, and eating a healthy diet to prevent complications of cancer treatment and recurrence [6]. Self-efficacy positively affects the QOL of patients with breast cancer by allowing them to actively cope with health issues related to breast cancer [14, 15] and engage in healthy self-care behaviors [13, 14, 16, 17]. Social support is also a major environmental factor that affects the QOL of patients with breast cancer. Social support is defined as the feeling of being protected or helped by others [21]. With sufficient social support, patients with breast cancer can actively cope with health problems [17, 18, 22] and develop a positive outlook on life [17]. Ultimately, social support improves the QOL of patients with breast cancer [16, 23].

Previous studies have focused primarily on the timing of cancer diagnosis or have examined patients who were receiving surgery or cancer treatment [12, 24]. Furthermore, the QOL of survivors varies depending on the time after the end of adjuvant treatment, but most previous studies on survivors have had limitations in that they identified QOL by simultaneously recruiting breast cancer patients at various points in time after the cancer treatment. The transitional survivorship phase is important because patients learn to transition into normal life during this time [6]. Survivorship focuses on the post-treatment health and QOL of individuals with cancer until end of life. The QOL during the post-treatment completion period has the greatest influence on cancer survivors’ health and long-term QOL. Healthcare professionals should provide interventions for improving QOL and health to patients who are undergoing this transition phase (that is, from being a patient to resuming normal life) [3–5]. Various studies should be conducted to examine the factors affecting QOL during this transition period; this will help in developing effective intervention programs. Therefore, we evaluated QOL among patients with breast cancer who were transitioning from their status as patients to a new status as survivors and identified the factors that affected their QOL.

## Materials and methods

### Study design and participants

This cross-sectional study identified determinants of QOL in women with breast cancer immediately following the completion of treatment. Of the patients with breast cancer who received surgery, adjuvant chemotherapy, and/or radiotherapy, 140 patients satisfied the inclusion criteria, understood the purpose of this study, and gave written consent to participate. The inclusion criteria were patients who: 1) were aged 19–64 years diagnosed with first primary, pathology-confirmed stage I-IIIA breast cancers; 2) completed initial primary treatments with surgery, radiation and/or chemotherapy within one month of enrolment into the study; 3) were allowed to receive endocrine therapy and/or HER2 targeted adjuvant therapy; 4) were without any mental problems such as depression, recurrence, or metastasis; and 5) who could communicate and complete a questionnaire.

Cohen’s sample size determination was used to calculate the sample size needed for multiple regression analysis with six independent variables given a moderate effect size of f^2^ = .15, significance level of α = .05, and power of 1 - β = 90% based on previous studies. The required sample size was calculated as 123; more than enough participants (n = 140) were recruited for this study.

### Instruments

#### Symptom experience

We used the Memorial Symptom Assessment Scale-Short Form (MSAS-SF) to measure the presence and severity of 32 physical and psychological symptoms [25]. The physical symptom distress score (PHYS) was determined via 12 questions, which addressed loss of appetite, lack of energy, pain, drowsiness, constipation, dry mouth, nausea, vomiting, changes in taste, weight loss, bloating, and dizziness. Each item is rated on a scale of 0 (no symptoms) to 4 (very painful symptoms). The psychological symptom distress score (PSYCH) is determined via six questions, which address depression, anxiety, nervousness, sensitivity, sleep disturbance, and decreased concentration. Each item is rated on a scale of 0 (no symptoms) to 4 (almost constantly). The total score is determined by taking the mean of all 32 questions and ranges from 0 to 4 points. A higher score denotes greater distress caused by the symptom experience. The MSAS-SF had a Cronbach’s α value of .91 in a previous study [26] and .94 in this study.

#### Self-efficacy

We used the Self-Efficacy Scale for Self-Management of Breast Cancer (SESSM-B) developed by Lee et al. [27] to measure self-efficacy related to self-management. The scale consists of 13 questions across five domains: coping with the demand for psychological information (three questions), managing side effects (three questions), maintaining a healthy lifestyle (three questions), complying with treatment (two questions), and sex life (two questions). Each question is rated on a five-point scale. Total scores range from 13 to 65 points. Higher scores indicate higher self-efficacy. The SESSM-B had a Cronbach’s α value of .81 in a previous study [27] and a value of .79 in this study.

#### Social support

We measured social support using the Korean version of the Interpersonal Support Evaluation List-12 (ISEL-12), originally developed by Cohen and colleagues [28] and adapted by Kim et al. [21]. The scale consists of 12 questions across three domains: tangible support received from others (four questions), appraisal support in times of difficulties (four questions), and belonging support from doing activities with others (four questions). The questions are rated on a four-point Likert scale with the following options: “not true at all,” “not true,” “true,” and “very true.” Six questions (1, 2, 7, 8, 11, and 12) are negatively worded. Positively worded questions are rated from 0 to 3 points, and the negatively worded questions are rated from 3 to 0 points. Total scores range from 0 to 36 points. Higher scores indicate receiving higher levels of social support. The Korean version of the ISEL-12 had a Cronbach’s α value of .86 in a previous study [21] and a value of .89 in this study.

#### Quality of life

We measured QOL using the Functional Assessment of Cancer Therapy-Breast (FACT-B) [29]. We used FACIT’s calculation method for the total and sub-scale scores. The FACT-B has a total of 37 questions, which are split among physical (PWB), social/family (SWB), emotional (EWB), functional well-being (FWB), and breast cancer-specific (BCS) subscales [30]. Each item is assessed on a scale ranging from 0 (not at all) to 4 (very much). The FACT-B total score is calculated by summing all five subscale scores, where a higher score denotes higher QOL. The FACT‐B has been commonly used in clinical trials, is simple to complete, and has been shown to be sensitive to performance status and disease extent [29]. The Korean version of the FACT-B had a Cronbach’s α of .90 at the time of its adoption [29] and .89 in this study.

### Data collection

Data were gathered from June to September 2018. The participants were recruited from a breast cancer outpatient clinic at a tertiary hospital. A trained research assistant identified eligible participants using medical records. On the day of the outpatient visit, a research assistant contacted them to explain the purpose of the study and conducted a survey using a structured questionnaire that was provided after obtaining written informed consent from patients interested in participating in the study. The patients were presented with a participant information sheet that outlined the specifics of the research and stressed the voluntary nature of their participation. To maintain confidentiality, each participant was provided a code known only to the researcher. The questionnaire required approximately 20 minutes to complete.

### Data analysis

Collected data were analyzed using the SPSS 25.0 program. Descriptive statistics were used to analyze sociodemographic and disease/treatment-related characteristics, symptom experience, self-efficacy, social support, and QOL. An independent t-test or a one-way ANOVA was used to determine the differences in QOL scores across sociodemographic and disease/treatment-related characteristics. Pearson’s correlation coefficients were used to analyze the relationships between symptom experience, self-efficacy, social support, and QOL. Hierarchical multiple regression was used to identify the factors associated with QOL. All variables that were associated with QOL in univariate analysis (*p* < 0.05) were entered into the hierarchical multiple regression models. Multicollinearity, residuals, and outliers were evaluated to determine whether the independent variables were suitable for regression analysis. Correlation coefficients were between .20 and .73, indicating that the predictor variables were independent of one another [31]. Tolerance was measured as between 0.43 and 0.93, and the variance inflation factor was measured as 1.07–2.31, indicating that no multicollinearity problems were present [31]. The assumptions of residual normality, homoscedasticity, and linearity were all satisfied. The Durbin-Watson statistic was 2.0, indicating no autocorrelation [31]. The maximum Cook’s distance for outlier evaluation was 0.11; as this did not exceed 1.0, there were no outliers [31]. Thus, the basic assumptions of multiple regression analysis were satisfied. In the hierarchical regression models, sociodemographic and disease/treatment-related characteristics were entered in Step 1. Symptom experience, self-efficacy, and social support were added in Step 2 as independent variables.

### Ethical considerations

Ethical considerations and approvals were obtained from the Institutional Review Board of Ajou University Hospital (AJIRB-SBR-SUR-18-122). The study was conducted according to the principles expressed in the Declaration of Helsinki. Prior to the data collection, we informed the participants about the study purpose, the risks and benefits of participation, the principles of voluntary participation, and their right to withdraw or refuse participation at any time without any consequences; they were also informed that the collected data would be used for research purposes only. We acquired written informed consent from all participants prior to data collection.

## Results

### Sociodemographic and disease/treatment-related characteristics of study participants

The mean age of the study participants was 48.9 years (SD = 8.0). Most were aged 45 years or younger (39.3%), completed high school or had less than high school completion (60.7%), were unemployed (67.1%), and were in the middle-income class (78.6%). Stage I cancer was the most common among the participants (57.9%), followed by Stage II (29.2%) and Stage III (12.9%) cancers. Of the participants, 60.0% received chemotherapy and 91.4% received radiotherapy. In addition, 89.3% were receiving hormone therapy. Significant differences in QOL were found according to perceived economic status (F = 4.61, *p* = .012) and chemotherapy (t = 2.41, *p* = .017; Table 1).

**Table 1:** Differences of quality of life according to sociodemographic and disease/treatment-related characteristics (N = 140).

Variables	Categories	n (%)	Quality of life			
			Mean±SD	t or F	*p*	Post-hoc analysis
Age (yr)	≤ 45	55(39.3)	98.17±20.37	0.56	.574	
	46–55	50(35.7)	98.36±20.78			
	> 55	35(25.0)	94.13±18.49			
Education	≤ High school	85(60.7)	97.21±18.98	-0.01	.991	
	≥ College	55(39.3)	97.25±21.67			
Employment	No	94(67.1)	95.33±19.38	-1.61	.109	
	Yes	46(32.9)	101.10±20.92			
Perceived economic status	High^a^	7(5.0)	95.71±13.85	4.61	.012	c<b,a
	Middle^b^	110(78.6)	99.65±18.64			
	Low^c^	23(16.4)	86.10±24.41			
Stage	Ⅰ	81(57.9)	99.98±20.16	1.85	.162	
	Ⅱ	41(29.2)	93.54±21.21			
	Ⅲ	18(12.9)	93.22±14.70			
Chemotherapy	Done	84(60.0)	93.96±20.79	2.41	.017	
	Not done	56(40.0)	102.13±17.86			
Radiation	Done	128(91.4)	97.71±16.65	-0.94	.350	
	Not done	12(8.6)	92.05±23.82			
Hormone therapy	Done	125(89.3)	97.35±20.64	-0.20	.842	
	Not done	15(10.7)	96.25±14.07			

Post-hoc analysis was performed using Scheffé test.

### Descriptive statistics of symptom experience, self-efficacy, social support, and quality of life

The mean QOL (FACT-B total score) was 97.23 (SD = 20.01). The study participants scored on average 21.89 (SD = 5.87) on the PWB, 17.10 (SD = 5.50) on the SWB, 17.32 (SD = 4.20) on the EWB, 17.39 (SD = 5.87) on the FWB, and 23.54 (SD = 6.80) on the BCS. The mean total symptom experience score was 0.95 (SD = 0.63). The mean score for physical symptoms was 0.97 (SD = 0.69), and that for psychological symptoms was 1.36 (SD = 0.92). The mean self-efficacy score was 49.50 (SD = 8.25), and the mean social support score was 23.31 (SD = 5.17). The study participants scored 8.64 (SD = 2.68) on appraisal support, 8.74 (SD = 2.44) on belonging support, and 5.94 (SD = 1.57) on tangible support (Table 2).

**Table 2:** Descriptive statistics of quality of life, symptom experience, self-efficacy, and social support of the study participants (N = 140).

Variables	Mean±SD	Minimum	Maximum	Range
Quality of life	97.23**±**20.01	35.00	138.00	0–148
Physical well being	21.89**±**5.16	3.00	28.00	0–28
Social well being	17.10**±**5.50	1.00	28.00	0–28
Emotional well being	17.32**±**4.20	6.00	24.00	0–24
Functional well being	17.39**±**5.87	3.00	28.00	0–28
Breast cancer subscale	23.54**±**6.80	4.00	37.00	0–40
Symptom experience	0.95**±**0.63	0.00	2.61	0–4
The physical symptoms	0.97**±**0.69	0.00	2.80	0–4
The psychological symptoms	1.36**±**0.92	0.00	3.73	0–4
Self-Efficacy	49.50**±**8.25	26.00	65.00	13–65
Social support	23.31**±**5.17	2.00	34.00	0–36
Appraisal support	8.64**±**2.68	0.00	12.00	0–12
Belonging support	8.74**±**2.44	0.00	12.00	0–12
Tangible support	5.94**±**1.57	2.00	10.00	0–12

### Relationships between symptom experience, self-efficacy, social support, and quality of life

A correlation analysis was performed to examine the relationships between symptom experience, self-efficacy, social support, and QOL. QOL decreased as scores increased on physical symptoms (r = -.684, *p* < .001) and psychological symptoms (r = -.725, *p* < .001). QOL increased as self-efficacy (r = .309, *p* = .001) and social support (r = .480, *p* < .001) increased. For the domains of social support, QOL increased as appraisal support (r = .456, *p* < .001) and belonging support (r = .487, *p* < .001) increased (Table 3).

**Table 3:** Relationships between symptom experience, self-efficacy, social support, and quality of life (N = 140).

Variables		Quality of life
		r (*p*)
Symptom experience		-.748 (< .001)
	The physical symptoms	-.684 (< .001)
	The psychological symptoms	-.725 (< .001)
Self-Efficacy		.309 (.001)
Social support		.480 (< .001)
	Appraisal support	.456 (< .001)
	Belonging support	.487 (< .001)
	Tangible support	.045 (.599)

### Determinants of the quality of life in the study participants

Hierarchical multiple regression was performed on the factors that significantly correlated with QOL (dependent variable) in the univariate analysis (Table 4). In the first regression (Step 1), perceived economic status (0 = low, 1 = middle and high) and chemotherapy (0 = not done, 1 = done), which were sociodemographic and disease/treatment-related characteristics that significantly correlated with QOL in the univariate analysis, were introduced. In the second regression (Step 2), symptom experience, self-efficacy, appraisal support, and belonging support were introduced to analyze the influence of these factors on QOL.

**Table 4:** Determinants of the quality of life in the study participants (N = 140).

Step	Variables	B	β	t	*p*	R	Adj. R^2^	ΔR^2^	F	*p*
**Step1**	Perceived economic status (low)	13.29	.25	3.04	.003	.32	.09		7.67	.001
	Chemotherapy (not done)	-8.09	-.20	-2.45	.015					
**Step2**	Perceived economic status (low)	4.06	.08	1.44	.153	.81	.64	.55	36.95	< .001
	Chemotherapy (not done)	-4.18	-.10	-1.96	.052					
	The physical Symptoms	-7.27	-.25	-3.27	.001					
	The psychological Symptoms	-9.18	-.42	-5.57	< .001					
	Self-efficacy	0.17	.07	1.20	.231					
	Appraisal support	0.84	.11	1.53	.128					
	Belonging support	1.20	.15	2.03	.044					

In the first stage (Step 1) of the two-stage hierarchical multiple regression, perceived economic status and chemotherapy explained a significant amount of the variance in QOL (*p* = .001, Adj R^2^ = .09). In the second stage (Step 2), physical symptoms, psychological symptoms, self-efficacy, appraisal support, and belonging support explained approximately 64% of the variance in QOL. Of these, physical symptoms (β = -.25, *p* = .001), psychological symptoms (β = -.42, *p* < .001), and belonging support (β = .15, *p* = .044) were significantly associated with QOL (Table 4).

## Discussion

With continuing improvements in early detection and treatment, the number of breast cancer survivors has been increasing steadily. The end of this turbulent immediate post-treatment period could be an appropriate starting point for assessing survivorship issues, as physical and psychosocial health begin to stabilize during this period. QOL during this period has emerged as a major influential factor for assessing the adaptation and health of cancer survivors [5]. Therefore, the QOL of patients with breast cancer who are undergoing the transitional survivorship phase has emerged as an important health issue [3, 7]. In this study, we examined the influence of symptom experience, self-efficacy, and social support on QOL of patients with breast cancer who had completed initial primary treatments within the previous month.

The mean total QOL score of the participants was 97.23. This score was slightly higher than the score reported by Ai et al. [8] for Chinese patients with breast cancer who received chemotherapy (mean = 93.9) and lower than the score reported by Brennan et al. [32] for Austrian patients with breast cancer who had just completed treatment (mean = 108.1). Lu et al. [33] compared QOL between Chinese and American patients with breast cancer who had just finished chemotherapy, finding that the Chinese patients had a similar score (mean = 94.7), to the patients in this study while the American patients scored higher (mean = 107.4).

These score differences can be attributed to the different age groups examined in the studies. Many studies have reported that patients with breast cancer in their 30s and 40s have lower QOL compared to those in their 50s and 60s [34]. In our study and Ai et al.’s study [8], in which QOL scores were relatively low, the mean age of participants was 48.9 and 48.1 years, respectively. In Lu et al.’s study [33], 85.9% of participants were Chinese women aged 55 years or less. However, in a study by Brennan et al. [32], in which QOL scores were relatively high, the mean age of participants was 56.0 years. In Lu et al.’s study [33], over half of the American cancer survivors were aged 55 years or older. Brennen et al. [32], who investigated differences in QOL among different age groups, reported that women in their 50s and 60s had higher QOL than those in their 30s and 40s; younger patients with breast cancer had poorer QOL. Patients with low QOL immediately after completing treatment have been shown to have difficulty returning to normal life [35]. In Korea, where over half of patients with breast cancer are in their 40s, continuous monitoring and interventions for QOL improvement after treatment are necessary.

In the univariate analysis, perceived economic status, chemotherapy, physical and psychological symptoms, self-efficacy, appraisal support, and belonging support significantly correlated with QOL. The influence of these factors on the QOL of patients with breast cancer who completed treatment was thus examined. These factors explained 64% of the variance in QOL. Of these factors, physical and psychological symptoms and belonging support significantly affected QOL. Psychological symptoms most significantly affected QOL. This result supports the previous findings of Miller et al. [36] and Park et al. [9], who reported that psychological symptoms were the most important factor affecting the QOL of patients with breast cancer. The current results are also consistent with the findings of Abu-Helalah et al. [37] and Akel et al. [38], who found that anxiety and depression significantly affected QOL. Psychological distress due to anxiety and depression directly affects the QOL of breast cancer survivors and may be highly associated with the long-term survival of these patients. Patients with breast cancer experience anxiety due to the fear of recurrence [9, 12, 39]. A psychosocial support system must be developed that helps patients with breast cancer who completed treatment to regain psychological stability.

In this study, physical symptoms significantly affected QOL, consistent with previous reports that treatment-related systemic side effects that occur after completion of treatment affect QOL [40] and physical symptoms such as fatigue and pain negatively affect QOL of breast cancer survivors [9]. While most physical symptoms caused by treatment and cancer are relieved over time, cognitive impairment, sleep disorder, sexual dysfunction, fever, pain, and fatigue may persist in patients with breast cancer who complete treatment. These symptoms can cause patients to develop negative emotions during their recovery and negatively affect psychological stability [41, 42]. Persistent physical symptoms can increase fatigue and hinder patients’ return to normal life, thereby reducing their QOL. Therefore, continuous and systematic symptom management is necessary for patients with breast cancer who are adapting to normal life.

Belonging support was found to significantly affect the QOL of patients with breast cancer who completed treatment. This is consistent with Kroenke et al. [34], who measured the impact of social life on QOL using the 19-item Medical Outcomes Study Social Support Survey and reported that positive social interactions significantly affect QOL. Sammarco and Konecny [43] and Leung et al. [23] reported that positive emotional response and informational support can improve QOL of breast cancer survivors who continue to experience high levels of stress after completing treatment. These findings emphasize the need for a social support system that provides patients with breast cancer with belonging and informational support after they complete treatment.

In this study, however, self-efficacy did not sufficiently explain the variance in QOL. This result conflicts with previous reports that self-efficacy is an important factor in QOL [44, 45]. Self-efficacy refers to one’s belief in one’s ability to successfully complete a task [20]. In the context of cancer survivors, self-efficacy refers to one’s belief in one’s ability to manage physical and psychosocial problems [46]. Previous studies suggested that self-efficacy increases over time after patients complete their treatment. The discrepancies in the correlation results for self-efficacy may be attributed to the patients in this study having just completed treatment before study participation. However, cancer-related self-efficacy improves QOL by allowing patients to actively manage treatment outcomes, seek social support, examine cancer-related symptoms, and change their lifestyles [13, 47]; therefore, self-efficacy is an essential aspect of rehabilitation of breast cancer survivors [13, 15, 47]. Further research on the association between self-efficacy and QOL is needed.

### Limitations

This study has several limitations. First, the results of this study cannot be generalized since it only surveyed patients with breast cancer who were treated at a single university. In addition, analysis of the changes in QOL experienced by the participants was limited due to the inherent limitations of cross-sectional survey studies. Furthermore, difference in symptom experience and QOL according to the treatment regimen was not assessed because this study did not investigate therapies for symptom relief such as complementary alternative therapies, herbal medicines, and so forth.

Nevertheless, a strength of this study is that it examined factors associated with the QOL of patients with breast cancer who are entering the transitional survivorship phase immediately after treatment, thereby creating a basis for interventional studies aimed at improving the QOL of such patients. Continued research on the factors that affect QOL is needed to develop effective nursing interventions for patients with breast cancer.

## Conclusion

In this study, physical and psychological symptoms and belonging support were found to significantly affect QOL. These results demonstrate that breast cancer survivors experience physical and psychological symptoms that adversely affect QOL, even after completing treatment. Therefore, psycho-educational interventions are needed to improve the QOL of breast cancer patients who are physically and psychologically vulnerable after the end of treatment. And it is necessary to provide a support system by identifying patients’ needs and their level of satisfaction with the social support they receive.

We propose conducting a follow-up study to evaluate QOL according to the level of interaction between the treatment regimen and symptom experience in patients with breast cancer immediately after the completion of primary treatment. In addition, a longitudinal study is needed to investigate changes in QOL and the factors that affect QOL during the transitional survivorship phase.
